# Rapid and sustained symptom reduction following psilocybin treatment for anxiety and depression in patients with life-threatening cancer: a randomized controlled trial

**DOI:** 10.1177/0269881116675512

**Published:** 2016-11-30

**Authors:** Stephen Ross, Anthony Bossis, Jeffrey Guss, Gabrielle Agin-Liebes, Tara Malone, Barry Cohen, Sarah E Mennenga, Alexander Belser, Krystallia Kalliontzi, James Babb, Zhe Su, Patricia Corby, Brian L Schmidt

**Affiliations:** 1 Department of Psychiatry, New York University School of Medicine, New York, NY, USA; 2 New York University College of Dentistry, Bluestone Center for Clinical Research, New York, NY, USA; 3 Department of Child and Adolescent Psychiatry, New York University School of Medicine, New York, NY, USA; 4 Department of Psychiatry, Bellevue Hospital Center, New York, USA; 5 NYU Langone Medical Center, New York, NY, USA; 6 New York University-Health and Hospitals Corporation (NYU-HHC) Clinical and Translational Science Institute, New York, NY, USA; 7 Department of Psychology, New York University, New York, NY, USA; 8 Department of Applied Psychology, New York University Steinhardt School of Culture, Education, and Human Development, New York, NY, USA; 9 Department of Radiology, New York University School of Medicine, New York, NY, USA; 10 Palo Alto University, Palo Alto, CA, USA

**Keywords:** Psilocybin, psychedelic, cancer, depression, anxiety, mystical experience

## Abstract

**Background:**

Clinically significant anxiety and depression are common in patients with cancer, and are associated with poor psychiatric and medical outcomes. Historical and recent research suggests a role for psilocybin to treat cancer-related anxiety and depression.

**Methods:**

In this double-blind, placebo-controlled, crossover trial, 29 patients with cancer-related anxiety and depression were randomly assigned and received treatment with single-dose psilocybin (0.3 mg/kg) or niacin, both in conjunction with psychotherapy. The primary outcomes were anxiety and depression assessed between groups prior to the crossover at 7 weeks.

**Results:**

Prior to the crossover, psilocybin produced immediate, substantial, and sustained improvements in anxiety and depression and led to decreases in cancer-related demoralization and hopelessness, improved spiritual wellbeing, and increased quality of life. At the 6.5-month follow-up, psilocybin was associated with enduring anxiolytic and anti-depressant effects (approximately 60–80% of participants continued with clinically significant reductions in depression or anxiety), sustained benefits in existential distress and quality of life, as well as improved attitudes towards death. The psilocybin-induced mystical experience mediated the therapeutic effect of psilocybin on anxiety and depression.

**Conclusions:**

In conjunction with psychotherapy, single moderate-dose psilocybin produced rapid, robust and enduring anxiolytic and anti-depressant effects in patients with cancer-related psychological distress.

Trial Registration: ClinicalTrials.gov Identifier: NCT00957359

## Introduction

Enduring clinically significant anxiety and/or depressive symptoms are common in patients with cancer, present in 30–40% of patients in hospital settings (Mitchell et al., 2011). These symptoms are associated with a variety of poor outcomes, including medication non-adherence, increased health care utilization, adverse medical outcomes, decreased quality of life, decreased social function, increased disability, hopelessness, increased pain, increased desire for hastened death, increased rates of suicide, and decreased survival rates (Arrieta et al., 2013; Brown et al., 2003; Jaiswal et al., 2014).

Although pharmacotherapeutic and psychosocial interventions are commonly used to treat anxiety and depression in cancer patients, their efficacy is mixed and limited (Grassi et al., 2014; NCCN, 2014). There are no US Food and Drug Administration approved pharmacotherapies for cancer-related psychological distress, the onset of clinical improvement with anti-depressants is delayed, relapse rates are high, and significant side effects compromise treatment adherence (Freedman, 2010; Li et al., 2012).

With a growing body of evidence linking higher levels of existential/spiritual wellbeing (in cancer patients) with improved quality of life and decreased depression/hopelessness/suicidality (Breitbart et al., 2000; McClain et al., 2003; Nelson et al., 2002), the need to develop effective therapeutic approaches to mitigate this domain of distress has become increasingly recognized within the disciplines of palliative care and psycho-oncology (emphasized within the last two decades by the Institute of Medicine, the World Health Organization, the National Comprehensive Cancer Network, the Joint Commission, the National Consensus Project, and the National Quality Forum) and improvement in these domains is now accepted as an integral component in the care of cancer patients (Puchalski, 2012). A number of manualized existentially oriented psychotherapies have been developed to address these existential/spiritual issues, with some empirical support from clinical trials (Lemay and Wilson, 2008), and several of these approaches were integrated into the therapy platform developed for this study. There are currently no pharmacotherapies or evidence-based combined pharmacological-psychosocial interventions to treat this particular type of distress and unmet clinical need in cancer patients (Breitbart et al., 2010).

Psilocybin, a tryptamine serotoninergic psychedelic, exerts its consciousness altering effects via 5HT2A agonism (Vollenweider and Kometer, 2010). It has a well-established physiological and psychological safety profile in human laboratory and clinical trial research (Johnson et al., 2008), is not known to be addictive and may have anti-addictive properties (Bogenschutz and Johnson, 2016; Krebs and Johansen, 2012; Ross, 2012). It can produce highly salient spiritual/mystical states of consciousness associated with enduring (months to years) positive changes in cognition, affect, behavior, and spirituality (Doblin, 1991; Griffiths et al., 2006, 2008, 2011; Pahnke, 1963). From the early 1960s to the early 1970s, clinical research utilizing the serotoninergic psychedelics, primarily lysergic acid diethylamide (LSD), to treat terminal cancer-related psychological and existential distress was conducted at major academic medical centers in the United States with a total of several hundred participants. These studies occurred largely in the context of open-label trials and showed improvements in the following symptom domains: anxiety, depression, fear of dying, quality of life, and pain (Grob et al., 2013; Grof et al., 1973; Kast, 1966; Kast and Collins, 1964; Pahnke et al., 1969).

Research into the use of hallucinogen treatment models for psycho-spiritual distress in advanced or terminal cancer ceased in the mid 1970s with the passage of the Controlled Substance Act of 1970, which placed all of the serotoninergic psychedelics into schedule I of the US Drug Enforcement Administration’s classification of regulated psychoactive substances.

Building upon hallucinogen research with cancer patients from over four decades ago, two recently published randomized controlled trials (RCTs) with serotoninergic psychedelics to treat cancer-related psychological distress, one using psilocybin in patients with advanced-stage cancer conducted at Harbor-UCLA (Grob et al., 2011) and the other using LSD in patients with a variety of life-threatening illnesses including but not limited to cancer diagnoses (Gasser et al., 2014), suggested acute and sustained treatment benefits. The University of California Los Angeles RCT in patients with advanced-stage cancer included a cohort of 12 participants and reported on the medical and psychiatric safety of administering low-dose psilocybin (0.2 mg/kg) in conjunction with psychotherapy, and revealed trends towards reduced depression and anxiety in the psilocybin group compared to the control condition (Grob et al., 2011).

In the present RCT, the primary hypothesis was that psilocybin, in conjunction with targeted psychotherapy, would significantly decrease anxiety and depression symptoms (compared to an active control, niacin, and the same dose of psychotherapy as the experimental group) in patients with life-threatening cancer diagnoses.

## Methods

### Study design and interventions

**Figure 1.**
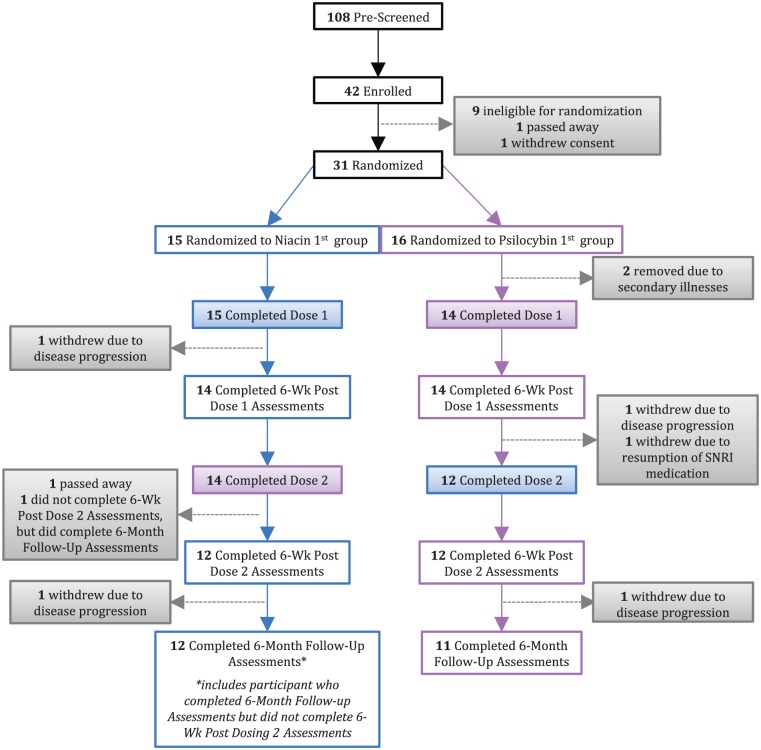
CONSORT diagram.

**Figure 2.**
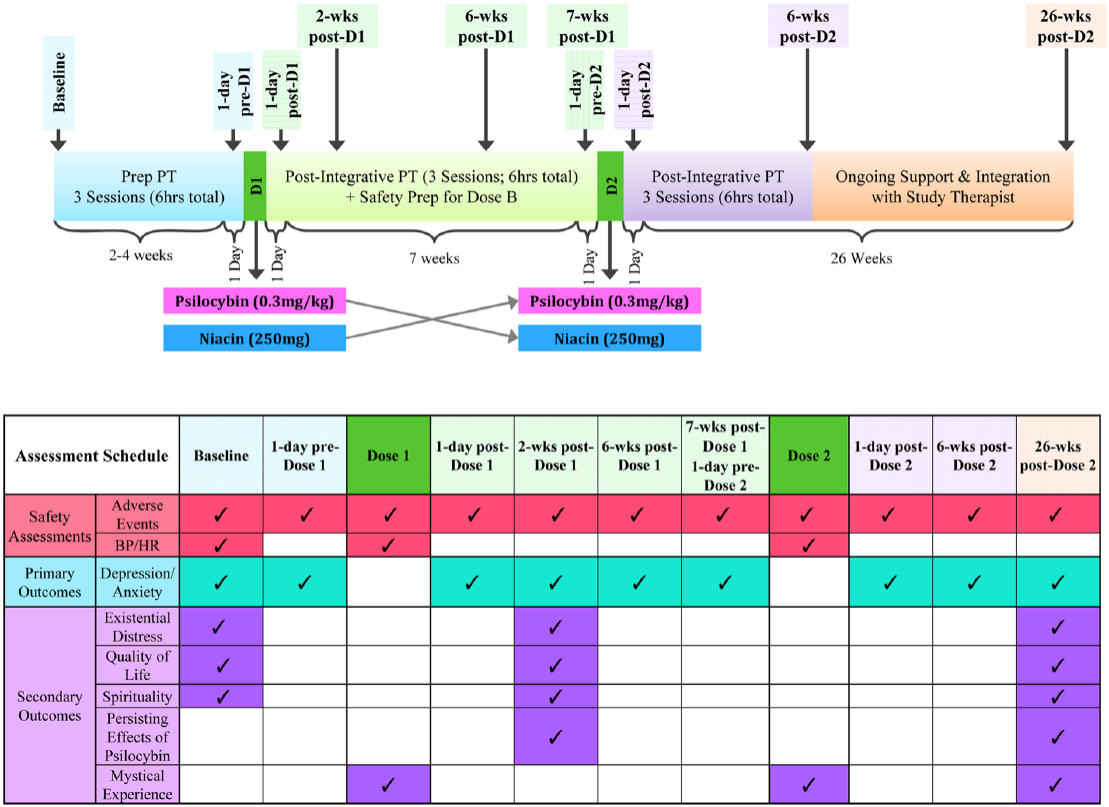
Interventions and assessments schedule. Temporal relationships between drug administration, psychosocial interventions, and assessments. Prep PT: preparatory psychotherapy; 1-day pre-D1: 1 day prior to dose 1; Dose 1: dosing session 1; 1-day post-D1: 1 day after dose 1; Post-integrative PT: post-integrative psychotherapy; 2-wks post-D1: 2 weeks after dose 1; 6-wks post-D1: 6 weeks after dose 1; Safety prep for D2: safety preparation for dosing dose 2; 1-day pre-D2: 1 day prior to dose 2; Dose 2: dosing session 2; 1-day post-D2: 1 day after dose 2; 6-wks post-D2: 6 weeks after dose 2; 26-wks post-D2: 2 weeks after dose 2.

This randomized, blinded, controlled, crossover, study was designed to investigate the efficacy of a single psilocybin dosing session (0.3 mg/kg) versus one dosing session of an active control (niacin 250 mg), administered in conjunction with psychotherapy, to treat clinically significant anxiety or depression in patients with life-threatening cancer (see Supplementary Methods for information on inclusion/exclusion criteria, blinding procedures, medication sessions and psychotherapy procedures). The trial employed a two-session, double-blind, crossover (7 weeks after administration of dose 1) design to compare groups. Participants were randomly assigned to two oral dosing session sequences: psilocybin (0.3 mg/kg) first then niacin (250 mg) second, or niacin (250 mg) first then psilocybin (0.3 mg/kg) second (Figures 1 and 2). Randomization did not stratify for any demographic (i.e. gender, race, spiritual/religious affiliation) or clinical characteristics (i.e. stage of cancer, prior hallucinogen use). Drug administration dose 1 (psilocybin or control) occurred 2–4 weeks (mean 18 days) after baseline assessments and the crossover occurred 7 weeks (mean 52 days) after dose 1, at which point drug administration dose 2 occurred. Data assessments occurred at baseline (2–4 weeks prior to dose 1), 1 day prior to dose 1, day of dose 1 (7 hours post-dose), 1 day after dose 1, 2 weeks after dose 1, 6 weeks after dose 1, 7 weeks after dose 1 (1 day prior to dose 2), day of dose 2 (7 hours post-dose), 1 day after dose 2, 6 weeks after dose 2, and 26 weeks after dose 2 (Figure 2). The total duration of study participation was approximately 9 months (mean 253 days). The primary outcome variables were anxiety and depression assessed prior to the crossover. Secondary outcome measures (assessed before and after the crossover) included assessments of existential distress, quality of life, and spirituality, as well as measures assessing immediate and sustained effects of psilocybin administration on subjective (e.g. mystical) experience, cognition, affect, spirituality, and behavior.

### Study sample and setting

Of 108 participants pre-screened, 42 gave informed consent and of these 29 patients were randomly assigned and received treatment with single-dose psilocybin or single-dose niacin control (Table 1 and Figure 1). The study was approved and monitored by the institutional review board of the New York University (NYU) School of Medicine. The majority of participants were recruited from a clinical cancer center at an academic medical facility (NYU Langone’s Perlmutter Cancer Center). Data were collected from 18 February 2009 to 22 October 2014 and the analysis was conducted from 3 November 2014 to 11 December 2015.

**Table 1.** Demographic and clinical characteristics of study participants.^a^

Characteristic	Categories	Psilocybin first		Niacin first		Total	
		*n*=14		*n*=15		*n*=29	
Sex	Female	7	50%	11	73%	18	62%
	Male	7	50%	4	27%	11	38%
Age; mean (SD)	Range 22–75	52 (15.03)		60.27 (9.45)		56.28 (12.93)	
Race	White/Caucasian	13	93%	13	87%	26	90%
	Black/African American	0	0%	0	0%	0	0%
	Hispanic/Latino	0	0%	0	0%	0	0%
	Asian	0	0%	0	0%	0	0%
	American Indian/Native American	0	0%	0	0%	0	0%
	Other	1	7%	2	13%	3	10%
Religious/spiritual beliefs	Atheist/agnostic	4	29%	10	67%	14	48%
	Jewish	4	29%	1	7%	5	17%
	Catholic	2	14%	0	0%	2	7%
	Other Christian	3	21%	1	7%	4	14%
	Other faith/tradition	1	7%	3	20%	4	14%
Site of cancer	Breast	4	29%	5	33%	9	31%
	Reproductive	3	21%	5	33%	8	28%
	Digestive cancers	3	21%	2	13%	5	17%
	Lymphoma/leukemia	2	14%	2	13%	4	14%
	Other types	2	14%	1	7%	3	10%
Stage of cancer	Stage IV	3	21%	7	47%	10	34%
	Stage III	4	29%	4	27%	8	28%
	Stage II	1	7%	4	27%	5	17%
	Stage I	5	36%	0	0%	5	17%
	Other	1	7%	0	0%	1	3%
SCID (DSM-IV) diagnosis^b^	Adjustment disorder w/anxiety and depressed mood, chronic	2	14%	6	40%	8	28%
	Adjustment disorder w/anxiety, chronic	10	71%	8	53%	18	62%
	Generalized anxiety disorder	2	14%	1	7%	3	10%
Hallucinogen use	No	7	50%	6	40%	13	45%
	Yes	7	50%	9	60%	16	55%
Employment status	Full-time employed	6	43%	5	33%	12	41%
	Part-time employed	2	14%	2	13%	4	14%
	Full-time student	1	7%	0	0%	1	3%
	Unemployed	2	14%	1	7%	2	7%
	Self-employed	1	7%	1	7%	2	7%
	Retired	0	0%	6	40%	6	21%
	Long-term disability	2	14%	0	0%	2	7%
Educational attainment	Grade 7–12 w/o graduating high school	1	7%	0	0%	1	3%
	Graduated HS or equivalent	0	0%	1	7%	1	3%
	Part college	1	7%	3	20%	4	14%
	Graduated 4-year college	5	36%	4	27%	9	31%
	Completed grad/professional school	7	50%	7	47%	14	48%
Marital status	Never married	5	36%	3	20%	8	28%
	Widowed	0	0%	2	13%	2	7%
	Cohabitation	2	14%	0	0%	2	7%
	Divorced	1	7%	3	20%	4	14%
	Married	6	43%	7	47%	13	45%
Living arrangements	Live with spouse/partner/family	11	79%	9	60%	20	69%
	Live alone	2	14%	6	40%	8	28%
	Other; lived with roommates	1	7%	0	0%	1	3%

a The two dose-sequence groups did not significantly differ on any demographic or clinical characteristic measures.

b Psychiatric classification was based on the structured clinical interview for the DSM-IV (SCID-IV).

Nearly two-thirds (59%) of participants had previously been treated with anti-depressant or anxiolytic medication, but none were on any psychotropics before study enrollment per inclusion/exclusion criteria.

Nearly two-thirds of participants (62%) had advanced cancers (stages III or IV). The types of cancer included: breast or reproductive (59%); gastrointestinal (17%); hematologic (14%); other (10%). In accordance with the study’s inclusion criteria, all participants carried an anxiety-related diagnosis per the severe combined immunodeficiency (SCID) (Diagnostic and Statistical Manual of Mental Disorders (DSM)-IV) with the majority meeting criteria for an adjustment disorder (26, 90%) and the rest for generalized anxiety disorder (three, 10%). Nearly two-thirds (59%) had previously been treated with anti-depressant or anxiolytic medication, but none were on any psychotropics at the time of study enrollment, per the inclusion/exclusion criteria.

### Assessments

#### Safety assessments

Adverse events (AEs) attributed to study medications (psilocybin, niacin) were monitored throughout the trial, including during and after medication administration sessions.

Cardiovascular measures were assessed during medication sessions. Systolic and diastolic blood pressure (BP) and heart rate (HR) were measured at the following time points during the medication dosing sessions: baseline, 30, 60, 90, 120, 180, 240, 300, 360 minutes post-dose administration.

#### Primary Outcome Measures

Clinical primary outcome measures (anxiety, depression) were assessed at baseline, 1 day prior to dose 1, 1 day after dose 1, 2-weeks after dose 1, 6 weeks after dose 1, 7 weeks after dose 1 (corresponding to 1 day prior to dose 2), 1 day after dose 2, 6 weeks after dose 2, and 26 weeks after dose 2: Hospital Anxiety and Depression Scale (HADS) (Zigmond and Snaith, 1983), self-rated subscales of anxiety (HADS anxiety or HAD A), depression (HADS depression or HAD D) and total (HADS total or HAD T) combined score in patients with physical health problems (e.g. cancer); Beck Depression Inventory (BDI) (Beck et al., 1988) self-report depression measure; Spielberger State-Trait Anxiety Inventory (STAI) (Spielberger, 1983) self-report measure of state (STAI state or STAI S) and trait (STAI trait or STAI T) anxiety.

#### Secondary outcome measures

Cancer-related existential distress (demoralization, hopelessness, attitudes and affect associated with disease progression and death) was assessed at baseline, 2 weeks post-dose 1, and 26 weeks post-dose 2: Demoralization (DEM) scale (Kissane et al., 2004), self-report measure of the cancer-related demoralization syndrome (e.g. despair, helplessness, existential distress such as loss of hope/meaning/purpose in life, a sense of ‘giving up’, desire for hastened death); Hopelessness Assessment and Illness (HAI) scale (Rosenfeld et al., 2011) self-report measure of hopelessness in advanced cancer; Death Anxiety Scale (DAS) (Templer, 1970) a self-report questionnaire assessing the level of death anxiety; Death Transcendence Scale (DTS) (VandeCreek, 1999) a self-report measure of positive attitudes and adaptations to the finitude of life.

Quality of life was assessed at baseline, 2 weeks post-dose 1 and 26 weeks post-dose 2: World Health Organization Qualify of Life scale, brief version (WHO-Bref) (WHO, 1994), self-report measure of quality of life in four domains (physical, psychological, social relationships, environment).

Spirituality was assessed at baseline, 2 weeks post-dose 1 and 26 weeks post-dose 2: Functional Assessment of Chronic Illness Therapy-Spiritual Well-Being (FACIT-SWB) (Brady et al., 1999) a self-report measure of spiritual wellbeing generating three scales: meaning/peace, faith, total spiritual wellbeing score. The meaning/peace scale assesses one’s sense of inner peace, meaning, and purpose in life and corresponds to the more existential components of religious or spiritual practice. The faith scale measures strength and comfort derived from one’s faith and emphasizes the more ritualized components of religious/spiritual practice.

Subjective drug effects/mystical experience was assessed at 7 hours after drug administration sessions and retrospectively at 26 weeks post-dose 2: the Mystical Experience Questionnaire (MEQ 30) (Barrett et al., 2015) is a self-report questionnaire that evaluates discrete mystical experiences induced by serotoninergic psychedelics and is sensitive to detecting psilocybin-induced mystical experiences (MacLean et al., 2012). In addition to an MEQ total score, the questionnaire generates four empirically derived factors: mystical; positive mood; transcendence of time and space; and ineffability. A retrospective version of the MEQ 30 (MEQ retrospective scale) was administered at 26 weeks post-dose 2. See Supplementary Methods section for more information on the MEQ 30 and for other measures of subjective drug effects/mystical experience measured 7 hours after drug administration sessions.

Persisting effects of psilocybin were assessed at 2 weeks post-dose 1 and 26 weeks post-dose 2: the Persisting Effects Questionnaire (PEQ), a self-report measure of changes in attitudes, moods, behaviors and spiritual experiences, sensitive to the longitudinal effects of psilocybin administration (Griffiths et al., 2006, 2008, 2011). All participants (including in both the psilocybin first and niacin first groups) were asked at 26 weeks after dose 2 to reflect on the meaningfulness, spiritual significance and changes in wellbeing relative to what they guessed was their psilocybin dosing experience (see Supplementary Methods secondary outcome measures).

See Supplementary Methods for other secondary outcome measures.

### Statistical analysis

Whenever multiple time points were included in the analysis for continuous measures, repeated measures regressions, from the mixed effect repeated measurement (MMRM) model, were performed in SAS PROC MIXED using an AR(1) covariance structure and fixed effects of group and time. Comparison *t*-tests from the MMRM analyses are reported for the primary and the continuous secondary outcome measures (see below).

**Figure 3.**
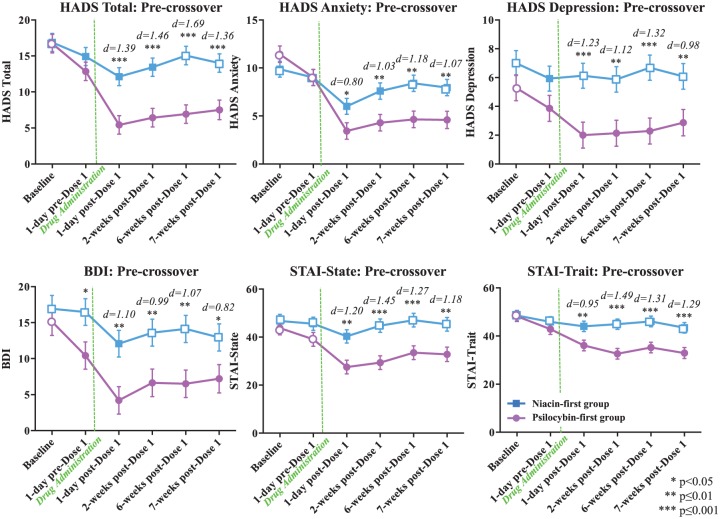
Primary outcome variables: cancer-related anxiety and depression (pre-crossover). Means (±SE) for primary outcome measures are shown in the two treatment groups at the following time points: baseline (psilocybin first *n*=14, niacin first *n*=15), 1 day pre-dose 1 (psilocybin first *n*=14, niacin first *n*=15), 1 day post-dose 1 (psilocybin first *n*=14, niacin first *n*=15), 2 weeks post-dose 1 (psilocybin first *n*=14, niacin first *n*=14), 6 weeks post-dose 1 (psilocybin first *n*=14, niacin first *n*=14), 7 weeks post-dose 1 (psilocybin first *n*=12, niacin first *n*=14). Asterisks indicate significance level of between-group *t*-tests. Effect sizes, represented as Cohen’s d, are shown above time points at which the treatment groups differ. Closed points represent significant within-group differences relative to scores at baseline.

**Figure 4.**
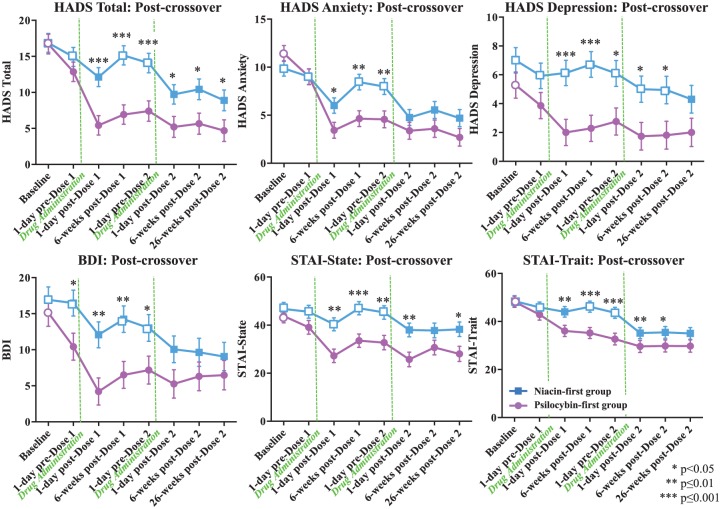
Primary outcome variables: cancer-related anxiety and depression (post-crossover). Means (±SE) for primary outcome measures are shown in the two treatment groups at the following time points: baseline (psilocybin first *n*=14, niacin first *n*=15), 1-day pre dose-1 (psilocybin first *n*=14, niacin first *n*=15), 1 day post-dose 1 (psilocybin first *n*=14, niacin first *n*=15), 6 weeks post-dose 1 (psilocybin first *n*=14, niacin first *n*=14), 7 weeks post-dose 1 (1 day pre-dose 2) (psilocybin first *n*=12, niacin first *n*=14), 1 day post-dose 2, 6 weeks post-dose 2 (psilocybin first *n*=12, niacin first *n*=11), 26 weeks post-dose 2 (psilocybin first *n*=11, niacin first *n*=12). Asterisks indicate significance level of between-group *t*-tests. Closed points represent significant within-group differences relative to scores at baseline.

For the primary outcome measures (anxiety, depression) in the two dosing sequences, planned between-group comparisons were made at the following time points: prior to the crossover at baseline, 1 day pre-dose 1, 1 day post-dose 1, 2 weeks post-dose 1, 6 weeks post-dose 1, 7 weeks post-dose 1 (corresponding to 1 day pre-dose 2) (Figure 3) and after the crossover at 1 day post-dose 2, 6 weeks post-dose 2, and 26 weeks post-dose 2 (Figure 4). Between-group effect sizes were calculated using Cohen’s d. Planned within-group comparison *t*-tests were conducted for each of the dosing sequences comparing the baseline to each of the following time points: 1 day pre-dose 1, 1 day post-dose 1, 2 weeks post-dose 1, 6 weeks post-dose 1, 7 weeks post-dose 1 (1 day pre-dose 2), 1 day post-dose 2, 6 weeks post-dose 2, 26 weeks post-dose 2 (Figures 3 and 4). Within-group effect sizes for the dosing sequences were calculated at each time point, compared to baseline, using Cohen’s d (Supplementary Table 1). To assess whether the magnitude of psilocybin-induced change in anxiety and depression differed across treatment groups, we compared change scores on the six primary outcome measures across each participant’s active (psilocybin) treatment session (from 1 day prior to psilocybin treatment to 1 day after psilocybin treatment) with one-way analysis of variance (ANOVA).

**Figure 5.**
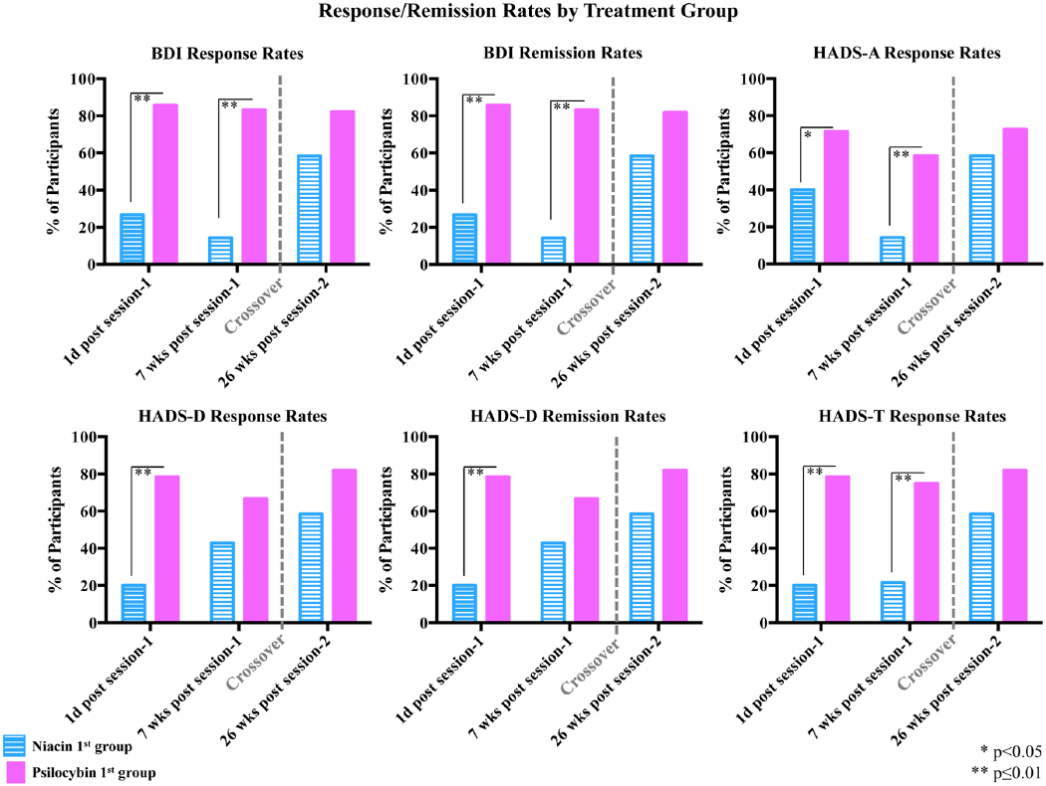
Percentage of participants with anti-depressant or anxiolytic response rates and anti-depressant symptom remission. Percentages of participants in each treatment group who met criteria for anti-depressant or anxiolytic response or anti-depressant symptom remission (BDI, HAD D) at 1 day post-dose 1 (psilocybin first *n*=14, niacin first *n*=15), 7 weeks post-dose 1 (psilocybin first *n*=12, niacin first *n*=14) and at 26 weeks post-dose 2 (psilocybin first *n*=11, niacin first *n*=12). Asterisks indicate significance level of between-group comparisons at each time point.

For primary outcome measures (HAD D, BDI, HAD A, HAD T) that have empirical support in defining anti-depressant or anxiolytic response, clinically significant responses rates were defined as a 50% or greater reduction in the measure at a particular assessment point relative to baseline. Anti-depressant symptom remission (HAD D, BDI) was defined as 50% or greater reduction in depressive symptoms plus HADS D ⩽7 (Hung et al., 2012) or BDI ⩽12 (Reeves et al., 2012; Riedel et al., 2010), respectively. Planned chi-square analyses were performed to compare the percentage of participants, in the psilocybin first versus the niacin first groups, who met criteria for anxiolytic or anti-depressant response, or anti-depressant remission (BDI, HAD D) at the following time points: 1 day post-dose 1, 7 weeks post-dose 1, and 26 weeks post-dose 2 (Figure 5).

For cardiovascular measures assessed during the medication sessions, repeated measures regressions, from the mixed effect repeat measurement (MMRM) model, were conducted in SAS PROC MIXED using an AR(1) covariance structure and fixed effects of time, drug (psilocybin vs. niacin) and group (niacin first vs. psilocybin first) collapsed across treatment order at time points: baseline, 30, 60, 90, 120, 180, 240, 300, 360 post-dosing (Supplementary Figure 1).

**Figure 6.**
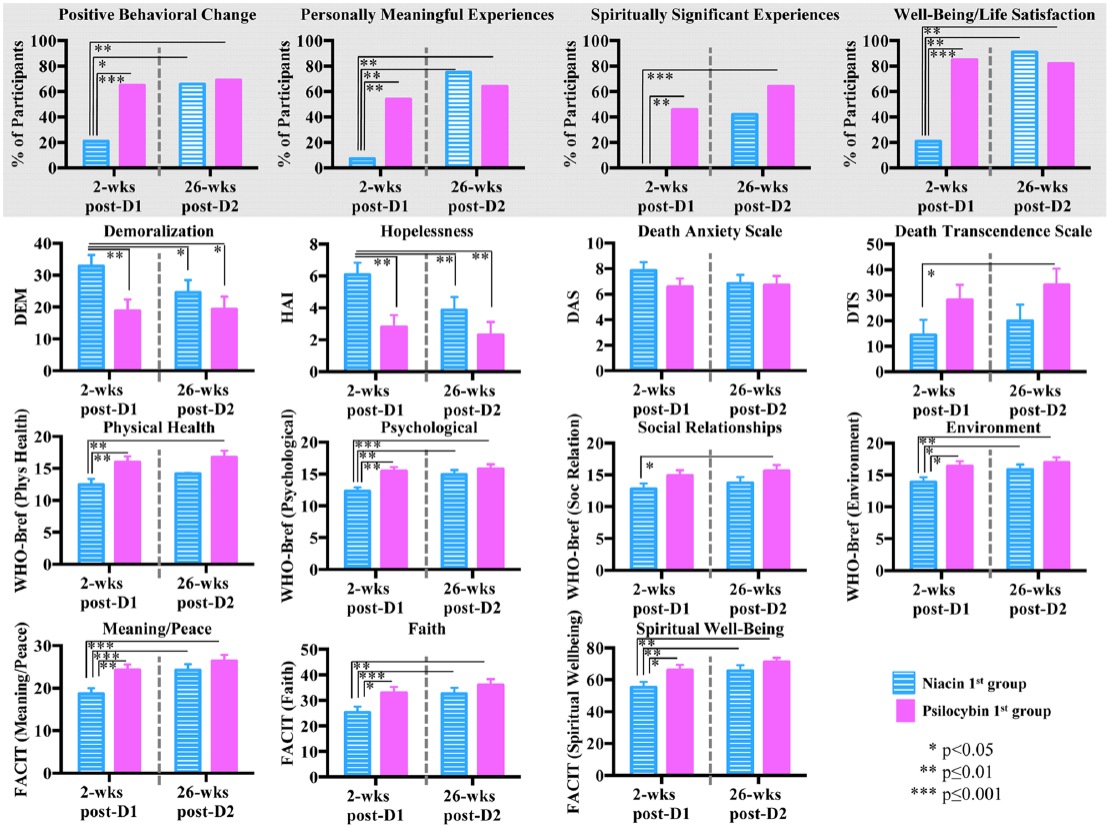
Secondary outcome measures: existential distress, quality of life, spirituality, persisting effects attributed to psilocybin administration. (Top) Percentage of participants that reported ‘among the top 5’ or ‘the single most’ personally meaningful and spiritually significant experiences, ‘moderate’, ‘strong’ or ‘extreme’ positive behavioral change, and ‘increased moderately’ or ‘increased very much’ wellbeing or life satisfaction on the Persisting Effects Questionnaire (PEQ). Asterisks indicate significance level of comparison to the niacin first group at 2 weeks post-dose 1. There were no significant differences between the psilocybin first group at 2 weeks post-dose 1 versus the psilocybin first group at 26 weeks post-dose 2. (Bottom) Secondary measures of cancer-related existential distress (DEM, HAI, DAS, DTS), quality of life (WHO-Bref) and spirituality (FACIT). Measures are shown at 2 weeks post-dose 1 (psilocybin first *n*=14, niacin first *n*=14) and at 26 weeks post-dose 2 (psilocybin first *n*=11, niacin first *n*=12); asterisks indicate significance level of comparison to the niacin first group at 2 weeks post-dose 1. There were no significant differences between the psilocybin first group at 2 weeks post-dose 1 versus the psilocybin first group at 26 weeks post-dose 2.

For the secondary outcome measures (cancer-related existential distress, quality of life, spirituality, persisting effects of psilocybin), planned between-group comparisons were conducted generating the following comparisons: 1. niacin first group 2 weeks post-dose 1 versus psilocybin first group 2 weeks post-dose 1; 2. niacin first group 2 weeks post-dose 1 versus niacin first group 26 weeks post-dose 2; 3. niacin first group 2 weeks post-dose 1 versus psilocybin first group 26 weeks post-dose 2; 4. psilocybin first group 2 weeks post-dose 1 versus psilocybin first group 26 weeks post-dose 2 (Figure 6 (bottom), Supplementary Table 2).

Ratings of persisting effects attributed to the medication sessions were expressed as proportions for four items (see Supplemental Methods): positive behavioral change; meaningfulness, spiritual significance, and increases in personal wellbeing. Planned chi-square analyses were performed: niacin first group at 2 weeks post-dose 1 and psilocybin first at 2 weeks post-dose 1, niacin first at 2 weeks post-dose 1 and psilocybin first at 26 weeks post-dose 2. McNemar tests were used to compare these proportions between the psilocybin first group at 2 weeks post-dose 1 and the psilocybin first group at 26 weeks post-dose 2 and between the niacin first group at 2 weeks post-dose 1 and the niacin first group at 26 weeks post-dose 2 (Figure 6 (top)).

**Figure 7.**
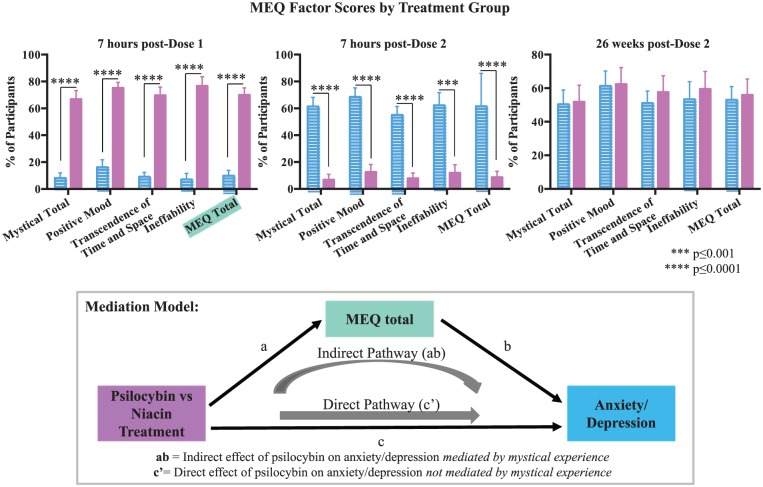
Subjective effects of psilocybin and relationship of mystical experience to clinical outcomes. (Top) Subjective effects as measured by the Mystical Experience Questionnaire (MEQ 30) in each treatment group at 7 hours post-session 1 (psilocybin first *n*=14, niacin first *n*=15), 7 hours post-session 2 (psilocybin first *n*=12, niacin first *n*=14), and 26 weeks post-dose 2 (psilocybin first *n*=11, niacin first *n*=12). Asterisks indicate significance level of between-group differences. (Bottom) Mediation model in which total scores on the MEQ transmit a portion of the effects of psilocybin versus niacin treatment on change in anxiety and depression is shown.

Subjective drug effects/mystical experiences were compared between groups using an independent sample *t*-test run in SAS at three time points: 7 hours post-medication administration in sessions 1 and 2; and at 26 weeks post-dose 2 (Figure 7 (top)). Anxiety and depression change scores for the primary outcome measures (ΔHADS T, ΔHADS A, ΔHADS D, ΔBDI, ΔSTAI S, ΔSTAI T) were calculated from baseline to 6 weeks post-dose 1 with either psilocybin or niacin. Spearman rank correlation coefficients were calculated between the change scores and participant ratings on the MEQ total at 7 hours post-dose 1 to assess the relationship between subjective mystical experience and change in clinical outcomes. Significant relationships were further examined using partial correlations to control for end of session participant-rated ‘intensity’ (item 98 from the HRS). In order to examine the mystical experience (using MEQ 30 scores) as a mediator of psilocybin versus niacin treatment on anxiety/depression outcomes, a bootstrap analysis was performed using the PROCESS macro (Hayes, 2013, Figure 7 (bottom)). The bootstrapping method is a non-parametric approach that does not assume a normal distribution of the mediated effect, is appropriate with small sample sizes, and was used to estimate 95% confidence intervals (CIs) for the mediation effect (Hayes, 2013). See Supplemental Methods.

See Supplementary Methods for additional statistical analysis.

## Results

### Demographics

As reported in Table 1, of the 29 participants who completed dose 1, the majority were Caucasian (90%) and women (62%). The average age was 56.3 (range 22–75) years. Approximately half of the participants reported some organized religious faith versus atheist/agnostic (52% vs. 48%) and slightly less than half reported no prior history of hallucinogen use (45%). Ninety per cent of participants met DSM-IV criteria for cancer-related adjustment disorder with anxious ± depressed features. The two dose-sequence groups did not significantly differ on demographic or clinical characteristic measures. No dichotomous factors (i.e. gender, prior hallucinogen use vs. none, spiritual faith/religion vs. none, early vs. late cancer stage) significantly interacted with the primary outcome measures in between-group comparisons.

### Safety assessments

#### Adverse events

There were no serious AEs, either medical or psychiatric, in the trial that were attributed to either psilocybin or niacin. Regarding psychiatric AEs, no pharmacological interventions (e.g. benzodiazepines, anti-psychotics) were needed during dosing sessions, no participants abused or became addicted to psilocybin, there were no cases of prolonged psychosis or hallucinogen persisting perceptual disorder (HPPD), and no participants required psychiatric hospitalization. In terms of AEs attributable to psilocybin, the most common medical AEs were non-clinically significant elevations in BP and HR (76%), headaches/migraines (28%), and nausea (14%); the most common psychiatric AEs were transient anxiety (17%) and transient psychotic-like symptoms (7%: one case of transient paranoid ideation and one case of transient thought disorder). The medical AEs (non-clinically significant elevations in BP and HR, headaches, nausea), and psychiatric AEs (transient anxiety, transient near-psychotic symptoms) attributable to psilocybin are all known AEs of psilocybin, were transient, tolerable, and consistent with prior trials of psilocybin administration in normal volunteers (Griffiths et al., 2006, 2008, 2011), and patients with terminal cancer (Grob et al., 2011).

#### Cardiovascular effects during dosing sessions

Compared to the control, psilocybin produced statistically significant differences in the following cardiovascular measures and time points: systolic BP: 60, 90, 120, 180, 240, 300 minutes; diastolic BP 60, 90, 120, 180 minutes; pulse: 90, 120 minutes (see Supplementary Figure 1). Cardiovascular effects with psilocybin generally peaked at 180 minutes post-dosing and decreased towards pre-drug levels over the remainder of the session. Regarding the psilocybin first group, peak mean systolic and diastolic BPs were 142/83 (both recorded at 180 minutes post-dosing), while peak mean HR for this group was 71 at 300 minutes post-dosing (see Supplementary Figure 1). There were no serious adverse cardiac events, consistent with psilocybin’s absence of cardiac toxicity when administered in controlled laboratory settings (Studerus et al., 2011). The medical safety, time course, and magnitude of effects on these cardiovascular measures were consistent with those observed in previous studies of psilocybin in healthy volunteers (Griffiths et al., 2006, 2011) and patients with advanced cancer (Grob et al., 2011).

### Primary outcomes

For each of the six primary outcome measures (HADS T, HADS A, HADS D, BDI, STAI S, STAI T), there were significant differences between the experimental and control groups (prior to the crossover at 7 weeks post-dose 1) with the psilocybin group (compared to the active control) demonstrating immediate, substantial, and sustained (up to 7 weeks post-dosing) clinical benefits in terms of reduction of anxiety and depression symptoms (Figure 3). The magnitude of differences between the psilocybin and control groups (Cohen’s d effect sizes) was large across the primary outcome measures, assessed at 1 day/2 weeks/6 weeks/7 weeks post-dose 1 (Figure 3).

Treatment groups did not differ in magnitude of change (e.g. 1 day before compared to 1 day after) across their respective psilocybin treatment sessions for any of the primary outcome measures (BDI: F_(1,26)_=1.88, *P*=0.18; HADS A: F_(1,26)_=2.59, *P*=0.12; HADS D: F_(1,26)_=0.90, *P*=0.35; HADS T: F_(1,26)_=2.63, *P*=0.12; STAI S: F_(1,26)_=1.10, *P*=0.30; STAI T: F_(1,26)_=0.58, *P*=0.45).

For all primary outcome measures, the psilocybin first group demonstrated significant within-group reductions (compared to baseline at each post-baseline assessment point) in anxiety and depression immediately after receiving psilocybin (Figures 3 and 4). These reductions remained significant at each time point, including the final point at 26 weeks post-dose 2 (approximately 8 months), post-psilocybin dosing. Prior to the crossover, the niacin first group demonstrated either no significant within-group reductions or a transient reduction that became non-significant prior to dose 2. For the majority (five/six) of the measures, the niacin first group demonstrated significant within-group reductions in anxiety and depression immediately after receiving the psilocybin dose (dosing session 2), and these statistically significant improvements persisted until the end of the study (approximately 6.5 months post-psilocybin dosing, 26 weeks post-dose 2, for this group).

Psilocybin produced immediate and enduring anxiolytic and anti-depressant response rates, as well as significant anti-depressant remission rates (measured by the HADS D and BDI) (Figure 5). For example, 7 weeks after dose 1, 83% of participants in the psilocybin first group (vs. 14% in the niacin first group) met criteria for anti-depressant response (with the BDI) and 58% (in the psilocybin first group) for anxiolytic response using the HAD A, compared to 14% in the niacin first group. At the 6.5-month follow-up (after both groups received psilocybin), anti-depressant or anxiolytic response rates were approximately 60–80% (Figure 5).

### Secondary outcomes

Figure 6 (bottom) shows the comparisons between dose-sequence groups on the following secondary outcome measures: cancer-related existential distress (demoralization, hopelessness, attitudes and affect associated with disease progression and death), quality of life, and spirituality. In the short-term (2 weeks post-dose 1), psilocybin (compared to control) produced decreases in cancer-related demoralization and hopelessness, while improving spiritual wellbeing and quality of life (physical, psychological, environmental domains). These effects were sustained at the final 6.5 month follow-up. Regarding anxiety and attitudes towards death, the data were mixed. In the short-term (2 weeks post-dose 1), psilocybin was not significantly associated with decreased death anxiety or increased death transcendence. However, at the 26-week post-dose 2 final follow-up assessment, while death anxiety (as measured by the DAS) continued to demonstrate no significant reductions, there was a significant improvement in attitudes and adaptations towards death (as measured by the DTS) in the psilocybin first group compared to the niacin first group (assessed at 2 weeks post-dose 1).

Supplementary Table 2 shows participant ratings of persisting effects attributed to the session experiences. As shown, prior to the crossover, psilocybin produced significantly greater ratings (compared to the niacin first group assessed at 2 weeks post-dose 1) of positive persisting effects on: attitudes about life and self, mood changes, social effects (e.g. increased altruism), behavior, and spirituality. After the crossover, these effects were sustained at the final 6.5-month follow-up. When all participants were asked (26 weeks post-session 2) to reflect on what they thought was their psilocybin session, 52% and 70% rated the psilocybin experience as the singular or top 5 most spiritually significant, or the singular or top 5 most personally meaningful experience of their entire lives, respectively; while 87% reported increased life satisfaction or wellbeing attributed to the experience (Figure 6 (top)).

### Mystical experience subjective effects and relationship of mystical experience to clinical outcomes

Compared to the control, psilocybin produced mystical-type experiences, consistent with prior trials of psilocybin administration in normal volunteers (Griffiths et al., 2006, 2008, 2011) and patients with terminal cancer (Grob et al., 2011) (Figure 7 (top)). Total mystical experience scores (MEQ 30) at the end of dose 1 (e.g. 7 hours post-drug administration) correlated with change scores (baseline to 6 weeks after dose 1) on four out of six primary outcome measures: HADS T (Spearman *r*=0.39; *P*=0.04); HADS A (Spearman *r*=0.36; *P*=0.07); HADS D (Spearman *r*=0.30; *P*=0.11); BDI (*r*=0.49; *P*=0.01); STAI S (*r*=0.42; *P*=0.03); STAI T (*r*=0.39; *P*=0.04).

Partial correlations to control for participant-rated intensity of drug effect (item 98 from the HRS) continued to demonstrate significant effects of total mystical experience scores (MEQ total) on the change scores (baseline to 6 weeks after dose 1) of the primary outcome measures in five of six measures assessed: HADS T (Spearman *r*=0.49; *P*=0.009); HADS A (Spearman *r*=0.46; *P*=0.01); HADS D (Spearman *r*=0.35; *P*=0.07); BDI (*r*=0.48; *P*=0.01); STAI S (*r*=0.42; *P*=0.03); STAI T (*r*=0.40; *P*=0.04).

MEQ total scores mediated (indirect effects) a significant portion of the effect of psilocybin versus niacin treatment on four out of six primary outcome measures with point estimates (ab) and bias corrected 95% CIs as follows: (HADS T (ab=0.46, SE=0.24, 95% CI 0.01–0.97), HADS D (ab=0.43, SE=0.32, 95% CI 0.01–1.23), BDI (ab=0.79, SE=0.26, 95% CI 0.23–1.29), and STAI S (ab=0.65, SE=0.25, 95% CI 0.13–1.16)] (Figure 7 (bottom)). Thus, the amount by which ΔHADS T, ΔHADS D, ΔBDI, and ΔSTAI S can be expected to increase through MEQ total as a result of psilocybin versus niacin treatment is 0.46, 0.43, 0.79 and 0.65, respectively.

For other analyses of secondary outcome measures, see Supplementary Results.

## Discussion

### Primary outcomes

Single moderate-dose psilocybin, in conjunction with psychotherapy, produced rapid, robust, and sustained clinical benefits in terms of reduction of anxiety and depression in patients with life-threatening cancer. This pharmacological finding is novel in psychiatry in terms of a single dose of a medication leading to immediate anti-depressant and anxiolytic effects with enduring (e.g. weeks to months) clinical benefits. Even though it is not possible to attribute causality of the experimental drug (in terms of sustained clinical benefit) after the crossover, the post-crossover data analyses of the two dosing sequences suggest that the clinical benefits, in terms of reduction of cancer-related anxiety and depression, of single-dose psilocybin (in conjunction with psychotherapy) may be sustained for longer than 7 weeks post-dosing, and that they may endure for as long as 8 months post-psilocybin dosing. The acute and sustained anti-depressant effects of psilocybin in this trial are consistent with a recently published open-label study of oral psilocybin treatment in patients with treatment-resistant depression (TRD) in which psilocybin (25 mg) was associated with 1 week and 3 months post-psilocybin anti-depressant effects (Carhart-Harris et al., 2016).

The within-group analyses for the primary outcome measures demonstrate that immediately after receiving psilocybin there is a marked reduction in anxiety and depression scores for both the psilocybin first and niacin first groups. Also, the magnitude of psilocybin-induced change across each participant’s active psilocybin treatment session did not differ across treatment group for any of the primary outcome measures. Together, this suggests that the pharmacological/psilocybin intervention produced rapid anti-depressant and anxiolytic clinical benefits. Both groups demonstrated significant clinical improvements in anxiety/depression from baseline relative to the final assessment. It is unclear from the data whether the sustained benefits in clinical outcomes were due to psilocybin alone or some interactive effect of psilocybin plus the targeted psychotherapy. Future research would be necessary to separate out the various therapeutic contributions of psilocybin versus psychotherapy.

Psilocybin was associated with substantial anti-depressant response rates (as high as approximately 80% at 6.5 months follow-up). There have been several meta-analyses of placebo controlled trials exploring the efficacy of anti-depressants in the treatment of cancer-related depression and they have generally failed to show a clear effect of anti-depressant treatment over placebo (Iovieno et al., 2011; Laoutidis and Mathiak, 2013; Ostuzzi et al., 2015). In a meta-analyses of anti-depressants for major depressive disorder in patients with comorbid medical disorders (including cancer), anti-depressants were more effective than placebo in some medical conditions (e.g. HIV/AIDS, post-stroke) but not in cancer patients, where the anti-depressants performed about as well as the approximately 40% placebo response rate (Iovieno et al., 2011).

### Secondary outcomes

Psilocybin decreased cancer-related demoralization (e.g. loss of meaning/hope/purpose, desire for hastened death) and hopelessness, while improving spiritual wellbeing, general life satisfaction, and quality of life. While a minority of patients with advanced or terminal cancer experience clinically relevant existential/spiritual distress, when it occurs its effects are highly consequential (e.g. decreased quality of life, increased depressive and anxiety symptoms, increased desire for hastened death, increased suicidal ideation and behaviors) (Puchalski, 2012) and improving spiritual wellbeing (e.g. through a pharmacological-psychosocial intervention) could serve as a buffer against these negative clinical outcomes.

Although affect/anxiety towards death did not improve in the short-term or longer-term follow-up period, psilocybin was associated with improved attitudes and adaptations to death at the 6.5-month follow-up. More research into this important therapeutic area is warranted.

Psilocybin experiences were reported as highly meaningful and spiritual, and associated with positive cognitive, affective, spiritual, and behavioral effects lasting weeks to months. This finding is consistent with prior research administering psilocybin to normal volunteers (Doblin, 1991; Griffiths et al., 2006, 2008, 2011; Pahnke, 1963).

### Safety/adverse events

There were no serious AEs, either medical or psychiatric, in the trial that were attributed to psilocybin. Since the early 1990s, approximately 2000 doses of psilocybin (ranging from low to high doses) have been safely administered to humans in the United States and Europe, in carefully controlled scientific settings, with no reports of any medical or psychiatric serious AEs, including no reported cases of prolonged psychosis or HPPD (Studerus et al., 2011). This finding is consistent with a US population (2001–2004 data from the National Survey on Drug Use and Health) based study that found no associations between lifetime use of any of the serotoninergic psychedelics (including psilocybin) and increased rates of mental illness (Krebs and Johansen, 2013). It is important to monitor closely for the emergence of transient difficult psychological states (e.g. anxiety, paranoia) in these trials and to manage them. Difficult experiences are not necessarily pathological and can be understood as part of the therapeutic process (e.g. working through cancer-related psychological or existential distress through challenging encounters or emotionally charged confrontations with cancer-related fearful imagery or symbolism) (Carbonaro et al., 2016).

### Limitations/generalizability

This trial was limited by a relatively small sample size, a non-nationally representative cancer patient population (e.g. 62% women, 90% Caucasian), which decreases generalizability, a crossover design that limited the interpretation of clinical benefits after the crossover, and the use of a control with limited blinding.

### Potential anxiolytic and anti-depressant mechanisms of psilocybin

#### Neurobiological mechanisms

There is evidence from animal research that serotoninergic psychedelics exert anxiolytic-like effects (Nichols, 2015). Several trials using animal models of anxiety demonstrated acute anxiolytic effects of the serotoninergic psychedelic 2,5-Dimethoxy-4-iodoamphetamine (DOI), a non-selective 5-HT2a/2c agonist (Nic Dhonnchadha et al., 2003; Ripoll et al., 2005, 2006). In two rodent studies, one with 5HT2A knockout mice (Weisstaub et al., 2006) and the other in rats with anti-sense-mediated 5HT2A downregulation (Cohen, 2005), the rodents displayed decreased anxiety-like behavior and in the trial with the 5HT2A knockout mice (Weisstaub et al., 2006), restoration of 5HT2A receptors in the pre-frontal cortex (PFC) re-established anxiety-like behaviors. Furthermore, in humans, fronto-limbic 5HT2A density has been correlated with anxiety symptoms (Frokjaer et al., 2008). Together, these data suggest that 5HT2A downregulation may explain some of the rapid and sustained anxiolytic effects of psilocybin (Vollenweider and Kometer, 2010).

There is growing evidence that the serotoninergic psychedelics produce rapid and sustained anti-depressant effects (Nichols, 2015). In two recently published open-label trials, one using a single dose of ayahuasca (Osorio et al., 2015) and the other using two doses of oral psilocybin (Carhart-Harris et al., 2016), acute and enduring anti-depressant effects were reported. In addition to these two open-label trials, there are several lines of evidence supporting using 5HT2A agonists to treat depression. In considering changes at the 5HT2A receptor as a potential mechanism of action: cortical 5HT2A receptor expression is increased in postmortem samples of patients with depression who display suicidality (Mendelson, 2000; Pandey et al., 2002; Shelton et al., 2009); depressed patients with elevated pessimism display increased PFC 5HT2A receptor binding compared to control participants (Bhagwagar et al., 2006; Meyer, 2012; Meyer et al., 2003); and sustained treatment with various anti-depressants (e.g. selective serotonin reuptake inhibitors, tricyclic anti-depressants) have been associated with a reduction of 5HT2A receptor density (Gomez-Gil et al., 2004; Yamauchi et al., 2006).

The glutamate system may explain some of the anti-depressant effects of psilocybin. In rodents, serotoninergic psychedelics enhance cortical glutamatergic transmission, especially in the medial PFC, and increase activation of cortical α-amino-3-hydroxy-5-methyl-4-isoxazolepropionic acid (AMPA) receptors (Aghajanian and Marek, 1997). In a trial in which rats received DOI, there was a significant increase in expression of brain-derived neurotrophic factor (BDNF) mRNA in neocortical areas (Vaidya et al., 1997). Increased AMPA activation and BDNF expression as biomarkers of anti-depressant effects are supported by: cortical AMPA activation is known to stimulate the expression of cortical BDNF (associated with neuronal growth, differentiation and synaptogenesis) (Hsu et al., 2015); decreased cortical BDNF is associated with major depression in humans (Duman, 2004); and cortical BDNF normalizes with anti-depressant treatment (Sen et al., 2008; Shimizu et al., 2003). Similarly, ketamine (the only other known acute and short-term sustained anti-depressant) is theorized to exert its anti-depressant effects via cortical AMPA activation (Zanos et al., 2016) and BDNF expression (Lepack et al., 2014). However, the anti-depressant effects of single-dose ketamine in patients with TRD typically last no more than several days up to 1–2 weeks, not several weeks to months (DeWilde et al., 2015).

Neuroimaging research with psilocybin is beginning to suggest potential anti-depressant mechanisms of action at the level of brain structure activity and network connectivity. Task-free functional magnetic resonance imaging research in normal volunteers under the influence of psilocybin has demonstrated decreased activity in the medial PFC and decreased connectivity within the default mode network (DMN) (Carhart-Harris et al., 2012, 2014). The former is significant because depressive symptoms have been associated with increased activity in the medial PFC (Drevets et al., 2008; Farb et al., 2011) and normalization of medial PFC activity has been demonstrated with anti-depressant treatment (Deakin et al., 2008; Holtzheimer and Mayberg, 2011; Kennedy et al., 2007); and the latter because patients with major depression (compared to controls) have demonstrated increased DMN connectivity (Berman et al., 2011, Grecius et al., 2007).

#### Psycho-spiritual mechanisms

Moderate-dose psilocybin occasioned mystical-type experiences in the cohort of cancer patients studied, and the intensity of the subjective mystical experience significantly mediated (e.g. suggestive of causality) clinical benefit (e.g. reduction in anxiety and depression symptoms) in the medium term (e.g. 6 weeks post-dose 1). This result matches with descriptive historical data from open-label LSD-assisted psychotherapy trials for psycho-spiritual distress associated with terminal cancer, in which the mystical experience was reported as being an integral part of the therapeutic effect (Grof and Halifax, 1977). It is further corroborated by recent open-label trials using psilocybin-assisted psychotherapy to treat tobacco addiction (Garcia-Romeu et al., 2014; Johnson et al., 2014) and alcoholism (Bogenschutz et al., 2015) showing significant correlations between the mystical experience and improved clinical outcomes.

This finding suggests a potential psycho-spiritual mechanism of action: the mystical state of consciousness. The mystical experience is likely to be one of several mediators that transmit the effect of psilocybin to changes in anxiety and/or depression. Further enquiry into how particular dimensions of the mystical experience relate to reductions in anxiety and/or depression in this population and others, and what factors best predict or promote mystical experiences, is warranted.

## Conclusions

In conclusion, single moderate-dose psilocybin (in conjunction with psychotherapy) was safely administered to a cohort of patients with cancer-related psychological distress (e.g. anxiety, depression). It produced rapid and sustained anxiolytic and anti-depressant effects (for at least 7 weeks but potentially as long as 8 months), decreased cancer-related existential distress, increased spiritual wellbeing and quality of life, and was associated with improved attitudes towards death. The psilocybin-induced mystical experience mediated the anxiolytic and anti-depressant effects of psilocybin. Psilocybin, administered in conjunction with appropriate psychotherapy, could become a novel pharmacological-psychosocial treatment modality for cancer-related psychological and existential distress. Further empirical research is needed definitively to establish its safety and efficacy.

## Supplementary Material

Supplementary material
