# Supplementary material for: Rapid and sustained symptom reduction following psilocybin treatment for anxiety and depression in patients with life-threatening cancer: a randomized controlled trial
Source: J Psychopharmacol. 2016 Nov 30;30(12):1165–80. doi: 10.1177/0269881116675512 (PMC5367551; doi:10.1177/0269881116675512)
Supplement: Supplementary material [file JOP675512_Appendix.pdf]

SUPPLEMENTARY APPENDIX

This appendix has been provided by the authors to give readers additional information about their work.

Supplement to: Ross, S., Bossis A., Guss, J. et al. Rapid and Sustained Symptom Reduction Following Psilocybin Treatment for Anxiety and Depression in Patients with Life-Threatening Cancer: A Randomized Controlled Trial

SUPPLEMENTARY APPENDIX

Rapid and Sustained Symptom Reduction Following Psilocybin Treatment for Anxiety and Depression in Patients with Life-Threatening Cancer: A Randomized Controlled Trial

Ross et al.

Supplemental Methods

Inclusion/Exclusion Criteria

Eligible participants were between 18 and 76 years of age, had a projected life expectancy of at least one year, and a primary diagnosis of Acute Stress Disorder, Generalized Anxiety Disorder (GAD), Anxiety Disorder Due to cancer, or Adjustment Disorder with Anxiety +/- Depression, as assessed by the Structural Clinical Interview for DSM-IV-TR Axis I Disorders-Patient Version. Participants were excluded if they had a score less than 8 on the Hospital Anxiety and Depression Scale (HADS). Medical exclusion criteria included epilepsy, renal disease, diabetes, abnormal liver function, and severe cardiovascular disease (i.e. congestive heart failure, uncontrolled hypertension). Psychiatric exclusion criteria included a personal or immediate family history of schizophrenia, bipolar disorder, delusional disorder, paranoid disorder, and schizoaffective disorder. Patients with a current substance use disorder were excluded. Study participants were free of concomitant psychotropic medications (e.g. anti-depressants) for two weeks prior to randomization and for the duration of the study. All patients underwent a physical examination and laboratory screening including hematologic and urine toxicology testing. An oncologist reviewed participant medical records and affirmed cancer diagnosis and life expectancy. Participants were not compensated for their time.

Initially, the study was designed to treat patients with ‘terminal,’ ‘stage IV’ cancer. Before study recruitment commenced and after further consultation with oncology and psycho-oncology colleagues, we determined that this language and criteria were overly restrictive and that patients with life threatening cancers did not necessarily need to be ‘terminal’ to have psychological distress (e.g. anxiety, depression) associated with the cancer. At approximately the mid-point of the trial (May 22, 2012), we further broadened the inclusion

criteria to include patients who were in 'remission' from their cancer (although still considered potentially life-threatening), but with significant psychological distress (e.g. anxiety, depression) associated with the cancer.

Randomization

A blocked randomization methodology was employed to equally randomize participants to either the experimental or control groups. Randomization occurred by dosing sequence and did not stratify for any demographic or clinical characteristics. Robert Norman PhD (Biostatistician at the Bluestone Center for Clinical Research, NYU College of Dentistry) generated the randomization list. Within each block, subjects were randomly assigned to active drug or placebo. In total, there were 8 blocks comprised of 4 participants. This list included subject numbers sequentially starting from 1 on upwards and next to each number there was a description (psilocybin or placebo). The list was always kept with the study documents in a secure location. This design ensured that half of the participants received psilocybin and that the other half received placebo 1st. The random allocation sequence was available only to administrative staff (Dr. Patricia Corby) at the Bluestone Center for Clinical Research. With each new participant, Dr. Corby (un-blinded) would consult the allocation sequence to determine what dosing sequence each particular participant would be randomized to. This information was given to un-blinded pharmacy staff to compound and prepare oral experimental drug or placebo in opaque (to preserve the blind), size 0 gelatin capsules.

Blinding Procedures: Double-blind dose conditions and instructions to participants and therapists

The trial compared the effects of single moderate-dose oral psilocybin (0.3 mg/kg) vs an active oral control (niacin 250 mg) in the session-1 condition before the cross-over, which occurred at approximately 7-weeks post session-1. The participants, study therapists, investigators, and raters were all blind to the drug administration conditions. Although challenging to adequately obscure the unique subjective effects of psilocybin, in an attempt to reduce participant and therapist expectancy biases, an active control (niacin) was used to mimic some of the effects of psilocybin administration (e.g. sense of warmth, arousal, tingling sensation). The participants and the therapists were told that on one occasion psilocybin would be administered and on the other occasion the control would be administered. At the end of each dosing session, the dyad treatment team (14 study staff

served as psychotherapists) recorded their guesses as to whether the participant received the psilocybin or the active placebo. These staff members guessed correctly in 28/29 participants (97%). Of note, the participants were not asked to record their guesses as to which drug they received on dosing session days.

Study Procedures for administration of moderate-dose psilocybin: Preparatory Psychotherapy, Medication Sessions, Post-dosing integrative Psychotherapy (see Figure 2)

Our conceptualization of the treatment intervention was that of a *medication-assisted psychotherapy* model. Our model was based on an historical model developed by Stanislav Grof that included the following components: Preparatory Psychotherapy, Medication Dosing Sessions, and Post-dosing Integrative Psychotherapy (Grof 1977; Grof 1973).

Therapist training: NYU Psychedelic Psychotherapy Training Program

Over the course of this research project, a total of sixteen clinicians were trained as study therapists, conducting preparation, dosing and integration sessions. The training program was developed and overseen by Dr. Jeffrey Guss. The didactic portion of the training process drew from principles of palliative care therapy, existential psychotherapy, psychoanalytic therapy, and transpersonal psychology. In addition, thorough grounding in the safety and clinical pharmacology of psilocybin was taught. Two therapists, usually one male and one female conducted all therapy sessions. Each therapist dyad underwent six confidential one-to-one sessions to build their alliance and exchange ideas about psychedelic therapy. Clinical case seminars, held with all study therapists present, allowed each co-therapy team to describe their treatment process, with group discussion following.

The study included 15 licensed mental health therapists over the duration of the trial: 6 psychiatrists; 2 psychologists; 4 oncology social workers; 1 oncology nurse; and 2 masters level counselors. All dyad teams had one of 3 lead therapists (Bossis, Guss, Ross). Each of the lead therapists had extensive clinical experience as psychotherapists, ranging from 20 to 35 years. Two (Guss, Ross) of the lead therapists were addiction psychiatrists and had extensive experience treating patients with severe/persistent mental illness (especially psychosis) and dangerous behavior towards self/others; one (Guss) was a psychoanalyst; and one (Bossis) was a

pain/palliative care psychologist with extensive experience in existentially-oriented and end-of-life psychotherapies.

Preparatory Psychotherapy

Following screening and study enrollment, participants were provided with three 2-hour psychotherapeutic preparation sessions with a therapy dyad (usually a male/female pair) for 6 total hours over a period of 2-4 weeks prior to the first treatment session in order to review the purpose and intention of participation in the study, treatment goals, and structure of the treatment sessions. Additional goals of the sessions: to establish rapport and trust between the participant and their two therapists; to review the participants' life histories as well as the meaning and nature of the psychological and existential distress associated with the cancer in relation to their lives; to review the nature and status of present relationships and concerns; to review the safety measures taken to minimize psychological adverse effects of psilocybin and the specific plan to manage (psychotherapeutically and pharmacologically) the occurrence of any significant such effects. Of note, participants were informed that two different classes (e.g. benzodiazepine, second generation anti-psychotic) of medications (e.g. diazepam, olanzapine) were available (in oral and parenteral preparations) to be used to manage major adverse psychological distress if necessary. The participants were reminded of this again on the dosing session days and the medications were stored inside of the dosing room for ease of use if necessary. Establishing safety prior to dosing sessions was vital and in addition to adequately reviewing all of the safety measures to minimize adverse psychological effects, if sufficient rapport was not established between the study participants and the dyad team, or if the dyad team felt for any reason that the participant was not psychologically prepared for the medication dosing session, the session would not occur or would be postponed until adequate safety could be established.

Medication Sessions

The medications were administered during two 8-hour treatment sessions, approximately 7-weeks apart. All research meetings and treatment sessions took place in an aesthetic living-room-like environment specifically designed for the study at the Bluestone Center for Clinical Research (BCCR) at the NYU College of

Dentistry. The study procedures followed historical (Grof 1977; Grof 1973) and modern (Johnson 2008) recommendations for administering moderate to high doses of a serotoninergic psychedelic. Psilocybin and niacin were administered in identically appearing opaque, size 0 gelatin capsules with approximately 180ml of water. On each of the two day-long medication treatment sessions, the couch in the room was made into a bed with sheets and blankets. An emesis basin was available in case of nausea and vomiting. The room included fresh flowers and fruit. Participants were encouraged to bring in items of personal significance and meaning (e.g. pictures of family members, other loved ones or pets; important spiritual or religious symbols). The study therapists assessed the participants to determine if dosing could safely proceed from both medical and psychiatric perspectives. Before dosing, the dyad therapy team and the participant joined hands (to convey unity and comfort to the participants and in a nod to the ritualized, indigenous, ceremonial, contained, and spiritual use of psychedelic plant medicines) and asked the participants to state their intention for the day. This latter part usually included some intention or hope on the part of the participant to find relief from cancer-related psychological and existential suffering. The participants were encouraged to lie comfortably and supine on a couch wearing eye shades as the default position post-dosing, to listen to pre-selected music (standardized to be the same for every participant and selected by the research team to temporally match the phenomenologic effects of psilocybin over its course of action), and to direct their attention to their internal experience. The study therapists remained with the participant throughout the entire 8-hour session and were available for psychological and medical support during the sessions. Towards the end of the dosing session, participants were encouraged to discuss the entirety of their subjective experience with the treatment team to consolidate the memory of the experience (especially given that these experiences can often be ephemeral and difficult to recall in detail, not dissimilar from dream states) and to begin the process of post-integrative psychotherapy. This was akin to doing psycholytic psychotherapy with a patient in a type of waking dream-like state. Medical (e.g. Blood pressure and heart rate) and psychiatric assessments were conducted at regular intervals to assess safety.

Post-dosing integrative Psychotherapy

Following each treatment session, participants met with their therapists for three 2-hour psychotherapy sessions (6 total hours), with the first session on the *day following* each medication session, to further consolidate the memory of the experience and to continue the process of psychological integration. For each participant, these three 2-hour post-integrative psychotherapy sessions occurred twice: the first occurred between dose 1 and dose 2 and the 2nd occurred over 6 weeks post-dose 2 **(Figure 2).** We constructed the post-dosing integrative psychotherapy component of the treatment from elements of the following types of psychotherapies which we thought would be key to interpret and make optimal therapeutic use of the psilocybin-induced subjective/mystical experiences and in particular included those with an evidence-based in cancer patients with psychological and/or existential distress: Supportive psychotherapy; cognitive-behavioral therapy; existentially oriented (i.e. psychotherapies developed specifically by psycho-oncologists to address existentially oriented issues that arise in patients with cancer and especially advanced-staged diagnoses); and psychodynamic/psychoanalytic. In a cochrane meta-analysis including 6 studies of patients with advanced stage cancer-related depressive symptoms, psychotherapy interventions (compared to standard treatment) were associated with significant decreases in depressive symptoms (Akechi et al., 2008). This review included: 4 studies of supportive psychotherapy, one with cognitive-behavioral therapy, and one with problem-solving therapy. In terms of the existential component to the psychotherapy, we drew from elements of evidence-based existentially oriented manualized psychotherapies (Lemay 2008), and in particular from 1)Meaning-making intervention (Creamer 1992, Lee et al., 2006); 2)Meaning-centered group psychotherapy (Frankl 1984; Breitbart 2003); 3)Dignity Psychotherapy (Chochinov et al., 2004); 4)Cognitive existential group therapy (Kissane et al., 2004); and 5)Re-creating your Life (Cole 1999). Even though we did not find any trials examining the use of psychodynamic/psychoanalytic therapy for cancer-related psychological or existential distress, we felt it important to include elements of this type of psychotherapy especially given the historical prominence of psychoanalytic theory as a key component of psycholytic therapies (i.e. entheogen enhanced psychoanalysis) (Grof 1976) and to make sense of the highly symbolic psychodynamic, archetypal, and mystical-type

experiences that are known to be part of the phenomenology of psilocybin-induced subjective states (Richards 1980). Additional follow-up meetings were held in person or over the telephone for six months.

Supplemental Methods: Secondary Outcome Measures

Other measures of psychological distress (e.g. anxiety, depression):

These measures were assessed at the following time points: Baseline, 1-day prior to session-1, 1-day after session-1, 2-weeks after session-1, 6-weeks after session-1, 7-weeks after session-1 (1-day prior to session-2), 1-day after session-2, 6-weeks after session-2, and 26-weeks after session-2.

The Profile of Mood States (**POMS**) (Cella 1987) is a self-report scale that assesses transient mood states. The POMS brief version was used in this trial. The analyses focused on items pertaining to depressive and anxious symptoms. The POMS individual items referring to ***depressive*** symptoms are: Bewildered, Weary, Gloomy, Muddled, Miserable, Discouraged, Blue, Sad, and Unhappy. The POMS items referring to ***anxious*** symptoms are: Uneasy and On Edge.

The Brief Symptom Inventory (**BSI**) (Derogatis 1993) is a 53-item self-report questionnaire used to assess a variety of psychological symptoms in patients undergoing psychiatric treatment, and generates 9 domains (somatization, obsession-compulsion, interpersonal sensitivity, depression, anxiety, hostility, phobic anxiety, paranoid ideation, psychoticism) and a global severity index. The analyses focused on the relevant subscales pertaining to anxious and depressed symptoms (***anxiety, depression)***, as well as the ***global severity index***.

Subjective drug effects/mystical experience

The Mystical Experience Questionnaire (**MEQ 30**) is based on Stace's description of classic mystical experiences and phenomenologically includes: unity (internal and external), transcendence of time and space, sacredness, positive mood, noetic quality (e.g. sense of 'ultimate reality'), paradoxicality, and ineffability (Stace

1960). Participants completed the 100-item *States of Consciousness Questionnaire* (Griffiths et al., 2006) rated on a 6-point scale [0=none, not at all; 1=so slight cannot decide; 2=slight; 3=moderate; 4=strong (equivalent in degree to any previous strong experience or expectation); 5=extreme (more than ever before in my life and stronger than 4)]. 30 items from this questionnaire generate the *Mystical Experience Questionnaire* (MEQ 30). In addition to an MEQ total score, the questionnaire generates 4 empirically-derived *factors*: **Mystical** (comprised of internal unity, external unity, noetic quality, and sacredness); **Positive Mood** (e.g. feelings of awe, joy, peace and tranquility); **Transcendence of Time and Space** (e.g. sense of being 'outside' of time, beyond past and future, in a realm of no space boundaries, sense of timelessness); **Ineffability** (e.g. experience cannot be adequately described in words) (MacLean 2012).

The other measures of subjective drug effect measures/mystical experience administered 7-hours after drug administration sessions were: The Hallucinogen Rating Scale (**HRS**), a self-report questionnaire designed to reflect the range of subjective experiences induced by the hallucinogen N,N-dimethyltryptamine and generates the following subscales: perception, cognition, affect, intensity, somaesthesia, and volition (Strassman et al., 1994); the 5 Dimension Altered States of Consciousness Profile (**5D-ASC**), a self-report questionnaire designed to measure altered states of consciousness occasioned by drug (e.g. psilocybin) or non-drug (e.g. sensory overload, starvation) stimuli, and generates three sub-scales: OSE (oceanic boundlessness corresponding to the mystical qualities of unity and transcendence of time/space); AIA (dysphoria, fear of ego dissolution corresponding to the 'bad trip' aspect described by users of hallucinogens); VUS (visionary restructuralization including items on synesthesia, illusions, visual pseudo-hallucination) (Dittrich 1998)

Persisting Effects of Psilocybin: Longitudinal changes in cognition, affect, behavior, spirituality

The Persisting Effects Questionnaire **(PEQ),** administered at 2-weeks post session-1 and 26-weeks post session-2, is a self-administered questionnaire that assesses changes in attitudes, moods, behaviors and spiritual experiences, and was developed to be sensitive to the longitudinal effects of psilocybin administration (Griffiths et al., 2011; Griffiths et al., 2008; Griffiths et al., 2006). It generates the following categories: positive attitudes

about life and/or self, negative attitudes about life and/or self, positive mood changes, negative mood changes, altruistic/positive social effects, antisocial/negative social effects, positive behavior changes, negative behavior changes, increased spirituality, decreased spirituality. The questionnaire also included three questions: (1) How personally meaningful was the experience (rated 1= no more than routine, everyday experiences; 2= similar to meaningful experiences that occur on average once or more a week; 3=similar to meaningful experiences that occur on average once a month; 4=similar to meaningful experiences that occur on average once a year; 5=similar to meaningful experiences that occur on average once every 5 years; 6=among the 10 most meaningful experiences of my life; 7=among the 5 most meaningful experiences of my life; and 8=the single most meaningful experience of my life?; (2) Indicate the degree to which the experience was spiritually significant to you (rated 1=not at all, 2=slightly, 3=moderately, 4=very much, 5=among the 5 most spiritually significant experiences of my life; and 6=the single most spiritually significant experience of my life). (3) Do you believe that the experience and your contemplation of that experience have led to change in your current sense of personal well-being or life satisfaction (rated +3=increased very much, +2=increased moderately, +1=increased slightly, 0=no change, −1=decreased slightly, −2=decreased moderately, and −3=decreased very much)?

Ratings of persisting effects attributed to the medication sessions were dichotomized for 4 categories by transforming them into 0's and 1's for four of the items. The rating categories transformed to 1's for those items were as follows: Positive behavioral change (‘moderate, ‘strong’ or ‘extreme’); Among the top 5 personally meaningful experiences of lifetime (‘among the top 5’ or ‘the single most’); Among the top 5 spiritually significant experiences of lifetime (‘among the top 5’ or ‘the single most’); and Increase in personal well-being or life-satisfaction (‘increased moderately’ or ‘increased very much’).

<u>Other Measures:</u> Other measures were assessed across the study that will be reported elsewhere. These measures include: subjective drug effects/mystical experience, affect, pain, spirituality, and spiritual/religious quality of life.

Additional Statistical Analysis

For other continuous measures of psychological distress (e.g. anxiety, depression derived from the BSI and POMS scales), repeated measures regressions from the MMRM model, were performed in SAS PROC MIXED using an AR(1) covariance structure and fixed effects of group and time. Planned between-group comparison t-tests from the MMRM analyses are reported at time points assessed prior to the cross-over: Baseline, 1-day pre session-1, 1-day post session-1, 2-weeks post session-1, 6-weeks post session-1, and 7-weeks post session-1 (**Figure S2**). Between-group effect sizes were calculated using Cohen's d.

Mediation analyses were performed to determine whether the observed relationships between psilocybin treatment and anxiety/depression were mediated by the mystical experience, as measured by MEQ total. The product of coefficients (ab) was calculated to represent the indirect effect of psilocybin treatment on anxiety/depression via the mystical experience using a bootstrap approach (10,000 samples) with the PROCESS macro (Hayes, 2013) in SAS. Preacher and Hayes' bootstrapping method is a non-parametric approach that does not assume a normal distribution of the mediated effect, and is appropriate to use with small sample sizes Bootstrapping uses repeated random sampling, with replacement, from the data set to calculate effect sizes of the direct pathway (c'), the indirect pathway (ab), and asymmetrical confidence intervals for these statistics (Hayes, 2013). Ninety-five percent asymmetrical confidence intervals (CIs) were calculated around ab and c' to determine our confidence that the value of these parameters in the sampled population is greater than zero, representing a significant effect. Here, the direct pathway (c') represents psilocybin effects on anxiety/depression independent of the mystical experience (MEQ total), and the indirect pathway (ab) represents psilocybin effects on anxiety/depression that are mediated by mystical experience (MEQ total) (**Figure 7 bottom**).

A two-way ANOVA was conducted to assess whether there were any significant differences between the 3-primary therapist groups (Bossis, Guss, Ross) and the primary outcome measures assessed at 7-weeks post session-1 and at the final study assessment point (26-weeks post session-2).

Supplemental Results

Other Anxiety and Depression Measures

The BSI and POMS outcome data are consistent with the results from the primary anxiety and depression outcome measures (e.g. HADS, BDI, STAI) (**see Figure 1 and Table S1**) in terms of psilocybin (versus placebo) producing rapid and enduring (at least 7-weeks post dosing) decreases in anxious and depressed symptoms (see **Figure S2**).

Primary Therapist/Treatment Groups and primary outcome measures

There were no significant differences between the 3 primary therapist/treatment groups and the primary outcome measures assessed at 7-weeks post dose-1 and 26-weeks post dose-2.

Supplemental References

Akechi T, Okuyama T, Onishi J, et al. (2008) Psychotherapy for depression among incurable cancer patients. *Cochrane Database of Systematic Reviews* 2:CD005537.

Breitbart W, Heller K (2003). Reframing hope: meaning-centered care for patients near the end of life. *Journal of Palliative Medicine* 6:979–988.

Cella DF, Jacobsen PB, Orav EJ, et al (1987). A brief POMS measure of distress for cancer patients. *J Chronic Dis* 40(10):939-942.

Chochinov HM, Hack T, Hassard T, et al. (2004). Dignity and psychotherapeutic considerations in end-of-life care. Journal of *Palliative Care* 20:134–142.

Cole B, Pergament K (1999). Re-creating your life: a spiritual/psychotherapeutic intervention for people diagnosed with cancer. *Psycho-Oncology* 8:395–407.

Creamer M, Burgess P, Pattison P (1992). Reaction to trauma: a cognitive processing model. *Journal of Abnormal Psychology* 101:452–459.

Derogatis LR (1993). *BSI Brief Symptom Inventory: Administration, Scoring, and Procedure Manual* (4th Ed.).Minneapolis, MN: National Computer Systems

Dittrich A (1998). The standardized psychometric assessment of altered states of consciousness (ASCs) in humans. *Pharmacopsychiatry* 31(Suppl 2):80-84

Frankl V (1984). Man's search for meaning. New York: Simon & Schuster.

Griffiths R, Johnson M, Richards W, et al. (2011) Psilocybin occasioned mystical-type experiences: immediate and persisting dose-related effects. *Psychopharmacology (Berl)* 218(4): 649-665.

Griffiths R, Richards W, Johnson M, et al. (2008) Mystical-type experiences occasioned by psilocybin mediate the attribution of personal meaning and spiritual significance 14 months later. *J Psychopharmacol* 22(6):621-632.

Griffiths R, Richards W, McCann U, et al. (2006) Psilocybin can occasion mystical-type experiences having

substantial and sustained personal meaning and spiritual significance. *Psychopharmacology (Berl)* 187(3):268-283; discussion 284-292.

Grob C, Danforth A, Chopra M, et al. (2011) Pilot study of psilocybin treatment for anxiety in patients with advanced-stage cancer. *Arch Gen Psychiatry* 68(1):71-78.

Grof S & Halifax J (1977). The Human Encounter with Death, New York, EP Dutton.

Grof S (1976). Realms of the Unconscious: Observations from LSD Research. New York: Viking Press.

Johnson MW, Richards W, Griffiths R (2008). Human hallucinogen research: guidelines for safety. *J Psychopharmacol* 22(6)**:**603-20.

Grof S, Goodman LE, Richards WA, et al. (1973) LSD-assisted psychotherapy in patients with terminal cancer. *Int Pharmacopsychiatry* 8**:**129-44.

Kissane DW, Love A, Hatton A, et al. (2004). Effect of cognitive-existential therapy on survival in early-stage breast cancer. *Journal of Clinical Oncology* 22:4255−4260.

Lee V, Cohen SR, Edgar L, et al. (2006). Meaning-making and psychological adjustment to cancer: development of an intervention and pilot results. *Oncology Nursing Forum* 33:291−302.

Lemay K, Wilson KG (2008). Treatment of existential distress in life threatening illness: a review of manualized interventions. *Clin Psychol Rev* 28(3):472-93.

Richards WA (1980). Psychedelic Drug-Assisted Psychotherapy with Persons Suffering fromTerminal Cancer. *Journal of Altered States of Consciousness* 5:309-319.

Strassman RJ, Qualls CR, Uhlenhuth EH, et al. (1994) Dose–response study of N, N-dimethyltryptamine in humans. II. Subjective effects and preliminary results of a new rating scale. *Arch Gen Psychiatry* 51:98–108

Supplemental Tables

Table S1: Primary Outcome Measures Within-Group Effect Sizes

Measure	Group	1-day pre Dose-1	1-day post Dose-1	6-weeks post Dose-1	1-day pre Dose-2	1-day post Dose-2	6-weeks post Dose-2	26-weeks post Dose-2
**HADS Total**	Psilocybin 1st	1.03	2.30	1.64	1.54	1.82	1.72	1.86
	Niacin 1st	0.51	0.97	0.31	0.45	1.11	1.06	1.22
**HADS Anxiety**	Psilocybin 1st	0.87	2.25	1.62	1.76	1.98	1.89	2.15
	Niacin 1st	0.31	1.09	0.35	0.47	1.23	1.11	1.26
**HADS Depression**	Psilocybin 1st	0.72	1.23	0.87	0.68	0.93	0.87	0.81
	Niacin 1st	0.54	0.32	0.09	0.24	0.51	0.55	0.66
**BDI**	Psilocybin 1st	1.05	1.82	1.12	1.07	1.24	1.05	1.03
	Niacin 1st	0.10	0.81	0.37	0.51	0.84	0.93	0.93
**STAI State**	Psilocybin 1st	0.49	1.33	0.72	0.81	1.28	0.92	1.13
	Niacin 1st	0.12	0.52	-0.02	0.10	0.60	0.66	0.60
**STAI Trait**	Psilocybin 1st	0.80	1.41	1.25	1.55	1.76	1.69	1.72
	Niacin 1st	0.40	0.52	0.24	0.48	0.89	1.07	1.13

Table S2: Persisting Effects Attributed to Psilocybin Administration

PEQ (% of max score)	Niacin 1st 2 wks post dose-1	Psilo 1st 2 wks post dose-1	Niacin 1st 26 wk post dose-2	Psilo 1st 26 wk post dose-2	Psilo attribution 26 wk post dose-2[a]
	N=14	N=14	N=12	N=11	N=23
Positive attitudes life	20.65 (3.51)	60.66 (3.51)***	61.49 (3.69)***	63.43 (3.80)***	
Negative attitudes life	29.69 (0.66)	26.15 (0.71)	26.15 (0.71)	22 (0.74)	
Positive attitudes self	20.13 (2.97)	53 (3.08)***	52.2 (3.12)***	52.63 (3.25)***	
Negative attitudes self	35.09 (0.68)	20.91 (0.71)	36.9 (0.72)	24 (0.75)	
Positive mood changes	16.67 (2.43)***	53.84 (2.52)***	48.09 (2.55)***	54.44 (2.66)***	
Negative mood changes	3.98 (0.60)	0.84 (0.62)	2.87 (0.65)	1.36 (0.67)	
Altruistic/positive social effects	19.36 (2.66)	49.58 (2.76)**	53.56 (2.86)***	52.49 (2.98)***	
Anti-social/negative social effects	3.49 (0.66)	0.84 (0.69)	3.27 (0.69)	0.71 (0.72)	
Positive Behavior changes[b]	21.4 (0.32)	64.6 (0.33)***	65.8 (0.34)***	68.8 (0.36)***	
Negative Behavior changes	4.2 (0.12)	3 (0.12)	2 (0.13)	0 (0)	
Increased Spirituality	17.46 (7.05)	52.65 (7.31)***	50.03 (7.37)***	57.42 (7.67)***	
Decreased Spirituality	0.68 (0.54)	1.47 (0.56)	1.99 (0.58)	1.21 (0.61)	
How personally meaningful was the experience? (max=8)	3.36 (0.44)	6.38 (0.46)***	6.43 (0.47)***	5.83 (0.49)***	
Top 5 most meaningful, including single most (%)[b]	7	54	75	64	70
How spiritually significant was the experience? (max=6)	2.07 (0.33)	4.54 (0.35)***	4.12 (0.36)***	4.54 (0.37)***	

**Top 5 most spiritually significant, including single[b] most (%)**	0	46	42	64	52
**Did the experience change your sense of well-being or life satisfaction? (max=6)**	0.71 (0.23)	2.38 (0.24)***	2.53 (0.25)***	2.24 (0.26)***	
**Increased well-being or life satisfaction moderately or very much (%)[b]**	21	85	91	82	87

Legend Text:

Numerical values in the columns represent the means (1 SEM) for persisting effects ratings: niacin-1st group at 2-weeks post dose-1, psilocybin-1st group at 2-weeks post dose-1, niacin-1st group at 26-weeks post dose-2, psilocybin-1st group at 26-weeks post dose-2.

a: All participants (including in both the psilocybin 1st and niacin 1st groups) were asked at 26-weeks post dose-2 to reflect on the meaningfulness, spiritual significance and changes in well-being relative to what they guessed was their psilocybin dosing experience, depending on how long after a participant received psilocybin (approximately 8-months for the psilocybin 1st group and approximately 6.5-months in the niacin 1st group).

b: See Figure 6 top panel: Persisting Effects Attributed to Psilocybin Administration. The percentage of participants endorsing specific answers on 4 items of the Persisting Effects Questionnaire (PEQ): Positive behavioral change (‘moderate, ‘strong’ or ‘extreme’); Among the top 5 personally meaningful experiences of lifetime (‘among the top 5’ or ‘the single most’); Among the top 5 spiritually significant experiences of lifetime (‘among the top 5’ or ‘the single most’); and Increase in personal well-being or life-satisfaction (‘increased moderately’ or ‘increased very much’).

Supplemental Figures

Figure S1: Cardiovascular Measures During the Medication Sessions

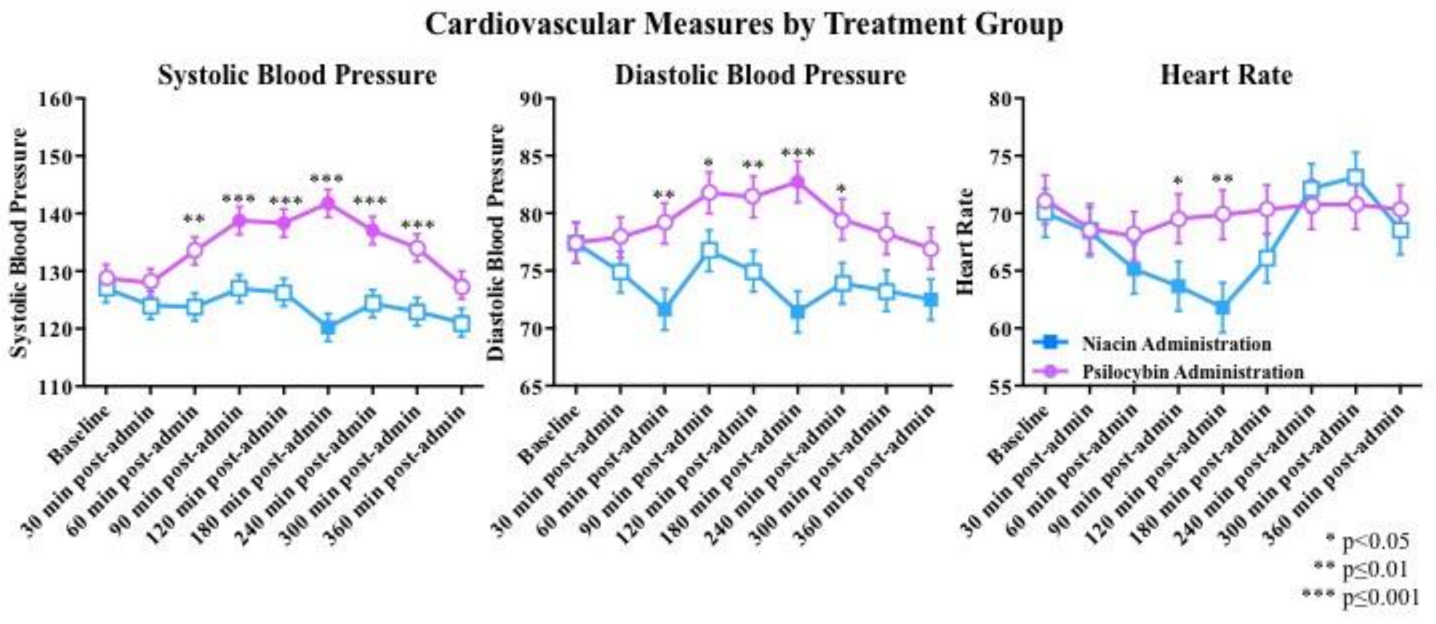

Legend Text:

Means (±SE) of each treatment group for active and inactive drug administration sessions, collapsed across treatment order, are presented. Asterisks indicated significant between-group differences; *closed* symbols represent significant within-group differences from Baseline.

Figure S2: Other Anxiety and Depression Measures (Pre-Crossover)

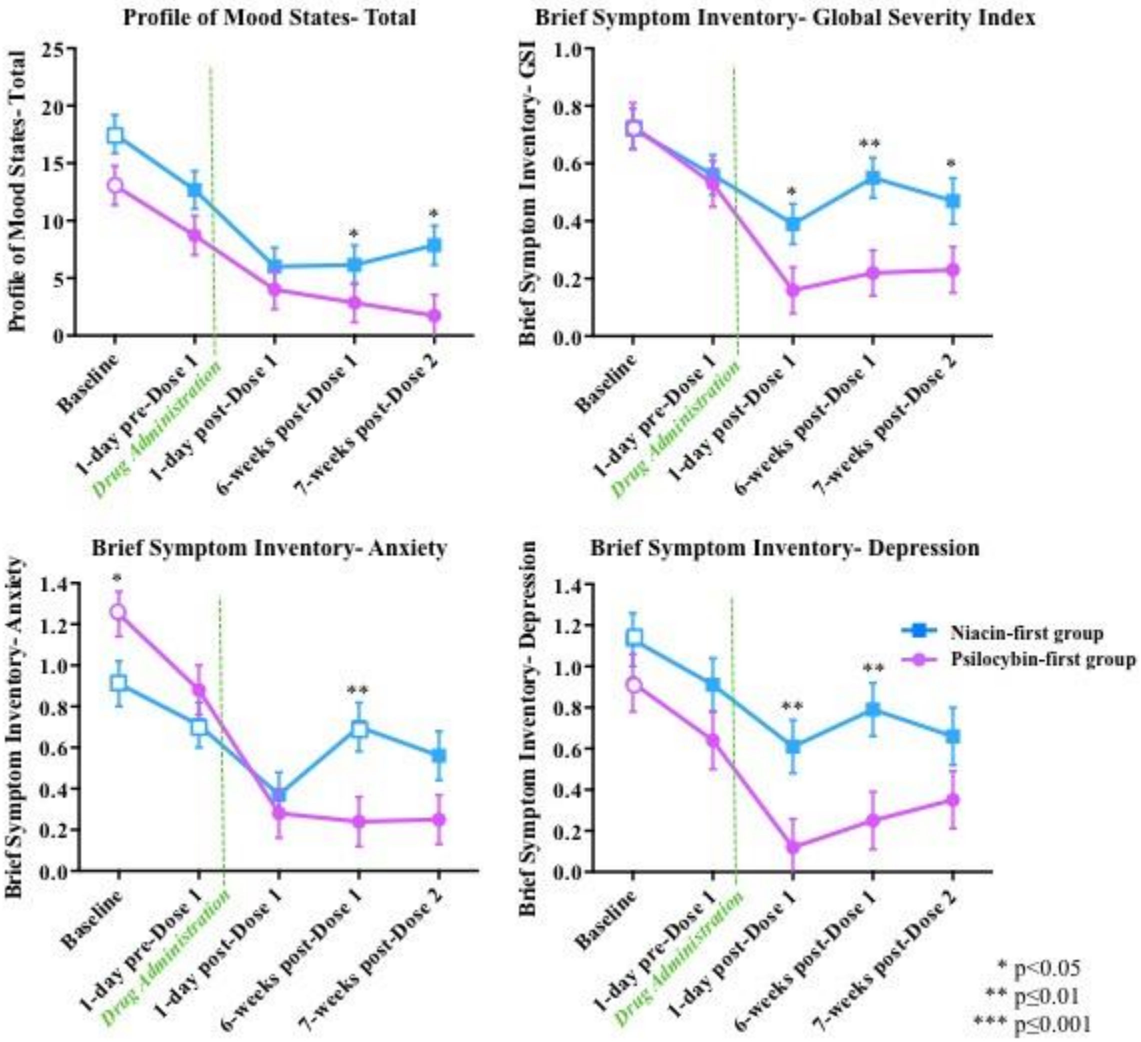

Legend Text: Means (± SE) for primary outcome measures in the two dose sequence groups. Between-group t-tests are reported at the following time points: baseline, 1-day pre session-1 (*Psilocybin-1st n=14, Niacin-1st n=15*), 1-day post session-1 (*Psilocybin-1st n=14, Niacin-1st n=15*), 2-weeks post session-1 (*Psilocybin-1st n=14, Niacin-1st n=14*), 6-weeks post session-1 (*Psilocybin-1st n=14, Niacin-1st n=14*), 7-weeks post session-1 (*Psilocybin-1st n=12, Niacin-1st n=14*). Asterisks indicated significant between-group differences; *closed* symbols represent significant within-group differences from Baseline.
